# HPLC-PDA-ESI-MS/MS Profiling and Anti-Biofilm Potential of *Eucalyptus* *sideroxylon* Flowers

**DOI:** 10.3390/antibiotics10070761

**Published:** 2021-06-23

**Authors:** Mona M. Okba, Riham A. El-Shiekh, Mohammed Abu-Elghait, Mansour Sobeh, Rehab M. S. Ashour

**Affiliations:** 1 Department of Pharmacognosy, Faculty of Pharmacy, Cairo University, Cairo 11562, Egypt; mona.morad@pharma.cu.edu.eg (M.M.O.); riham.adel@pharma.cu.edu.eg (R.A.E.-S.); 2 Department of Botany and Microbiology, Faculty of Science, Al-azhar University, Cairo 11884, Egypt; 3 AgroBioSciences Research Division, Mohammed VI Polytechnic University, Ben-Guerir 43150, Morocco; mansour.sobeh@um6p.ma

**Keywords:** *Eucalyptus sideroxylon*, LC-MS/MS, biofilm formation, multidrug-resistant bacterial infections

## Abstract

The development of multidrug-resistant bacterial strains is a worldwide emerging problem that needs a global solution. Exploring new natural antibiofilm agents is one of the most important alternative therapies in combating bacterial infections. This study aimed at testing the antimicrobial potential of *Eucalyptus sideroxylon* flowers extract (ESFE) against *Bacillus subtilis*, *Staphylococcus aureus*, *Escherichia coli*, *Pseudomonas aeruginosa* and *Candida albicans* prior to testing the antibiofilm activity against *S. aureus*, *P. aeruginosa* and *C. albicans*. ESFE demonstrated antimicrobial activity and promising inhibition activity against methicillin-resistant *S. aureus* (MRSA) biofilm formation up to 95.9% (*p* < 0.05) at a concentration of 0.05 mg/mL and eradicated *C. albicans* biofilm formation up to 71.2% (*p* < 0.05) at a concentration of 0.7 mg/mL. LC-MS analysis allowed the tentative identification of eighty-three secondary metabolites: 21 phloroglucinol, 18 terpenes, 16 flavonoids, 7 oleuropeic acid derivatives, 7 ellagic acid derivatives, 6 gallic acid derivatives, 3 phenolic acids, 3 fatty acids and 2 miscellaneous. In conclusion, *E. sideroxylon* is a rich source of effective constituents that promote its valorization as a promising candidate in the management of multidrug-resistant bacterial infections.

## 1. Introduction

Several pathogenic bacteria and fungi form a polymeric matrix called a biofilm. The latter increases the resistance of its aggregated cells to different groups of antibiotics and the host’s immune system elements by preventing their penetration through the bacterial surfaces [1,2]. A biofilm complex contains several molecules, such as extracellular DNA, lipids, proteins, and polysaccharides, which get absorbed into the implants and biotic surfaces to provide the initial attachment to the bacterial cell in order to succeed in the infection process [3].

The regulation of biofilm formation is considered one of the effective solutions against the worldwide emerging problem of increased bacterial resistance to antimicrobials [4]. Plant extracts have been reported to regulate biofilm formation and inhibit quorum sensing (QS) [5]. Various studies have explored different natural secondary metabolites to prevent the formation of biofilms in Gram-negative and Gram-positive bacteria, in addition to the dimorphic pathogenic yeast [4,6,7,8].

*Eucalyptus* is a diverse genus, with about 800 species and subspecies, of Myrtaceae flowering shrubs and trees. *Eucalyptus* is native to Australia and is widely distributed all over the world. It represents the second most widely spread plant genus [9]. *Eucalyptus* species were successfully introduced in many countries due to their commercial, medicinal and ornamental uses. *Eucalyptus* species are reported to be used traditionally in wound healing and in the treatment of fungal infections [10]. However, there is a lack of enough scientific data to support the mechanism by which *Eucalyptus* species can be used as antimicrobial agents.

*Eucalyptus sideroxylon* Cunn. ex Woolls is known as iron wood, mugga, and red-iron bark [11,12]. It has been previously tested for its effective use as alternative medicine in treating various bacterial and fungal infections [13,14]. Several secondary metabolites have been characterized from the plant, including phloroglucinols, flavonoids, tannins, oleuropeic acid glucose esters, sterols and triterpenes [15,16]. Our team previously identified 13 phloroglucinols in an extract of its leaves [15]. In an attempt to localize and quantify the phloroglucinols in different *Eucalyptus* species, it was found that the *E. sideroxylon* flowers’ tendency to accumulate phloroglucinols is about 5 times compared to the leaves. This was the sole study that traced *E. sideroxylon* flower phloroglucinols [17]. Apart from the essential oil, this is the first detailed chemical profile of *E. sideroxylon* flowers.

In the current work, the anti-biofilm potential of *E. sideroxylon* flowers against two strong biofilm forming bacteria (*Pseudomonas aeruginosa* and *Staphylococcus aureus*) in addition to the pathogenic yeast *Candida albicans* was investigated. The antimicrobial activities against other pathogens were also tested. A complete map of the *E. sideroxylon* flower’s secondary metabolites was also studied using LC-MS/MS.

## 2. Material and Methods

### 2.1. Plant Material and Extraction

*Eucalyptus sideroxylon* Cunn. ex Woolls flowers were collected in May 2018, from El-Kobba Palace, Cairo, Egypt. A voucher specimen (No. 05.06.19.I) was deposited in the museum of Pharmacognosy Department, Faculty of Pharmacy, Cairo University [15,16]. The air-dried flowers were ground, extracted by maceration in methanol and filtered. The combined methanol extract was evaporated under reduced pressure at temperature not exceeding 50 °C till dryness, yielding the crude *E. sideroxylon* flowers extract (ESFE).

### 2.2. Strains and Culture Conditions

For testing the antimicrobial and antibiofilm potentials, *Staphylococcus aureus* (ACL51) clinical strain, which is a potent biofilm-producing isolate that was subjected to 16SrRNA analysis, was used. The susceptibility profile of this strain was investigated using the VITEK^®^2 automated system (BioMerieux, Marcy-l’Étoile, France) and denoted as the methicillin-resistant *Staphylococcus aureus* (MRSA) strain. In addition, a coded methicillin-sensitive *Staphylococcus aureus* MSSA (ATCC 29213) strain as well as *Bacillus subtilis* (ATCC 6051), *Pseudomonas aeruginosa* (ATCC 27853), *Escherichia coli* (ATCC 35218) and *Candida albicans* (ATCC 90028) strains were used in this study.

### 2.3. Anti-Microbial Assay

The antimicrobial activity of ESFE was determined by Agar diffusion assay [18,19]. Muller-Hinton agar (MHA) (Oxoid, Hampshire, UK) and Tryptic soy agar (TSA) (Oxoid, Hampshire, UK) media were used to evaluate the growth of the tested bacterial and yeast strains. Microbial strains were cultured in TSA media by streaking and incubated for 24 h at 37 °C. Then after, 2–5 single colonies of the growth were suspended in melted MHA media and cooled to 50 °C with a density of 1.5 × 10^8^ CFU/mL (turbidity of 0.5 McFarland standards). They were then equally distributed into Petri dishes and left to solidify. A well with a diameter of 8 mm was punched with a sterile cork-borer, and 100 μL ESFE dissolved in 5% DMSO (final concentration in the well) was introduced into the well. The agar plates were incubated at 37 °C for 24 h. After incubation, the growth inhibition zone was determined using a digital caliper. The diameters of the zones were recorded in mm. The results were recorded according to CLSI guidelines [20].

### 2.4. Determination of the Minimum Inhibitory Concentration (MIC) and Minimum Bactericidal Concentration (MBC)

Microdilution assay using microtiter plates (MTP) was employed to test the MIC of ESFE against the tested organisms according to standard methods [20,21], with minor modifications. Two-fold serial dilutions of ESFE in DMSO (1% final concentration in the well) from 25 to 0.09 mg/mL *w*/*v* were distributed in a 96-well microtiter plate (SPL Life Sciences Co., Ltd, Pocheon-si, South Korea) and mixed with the specific microbial culture inoculated with the test organisms from 1:100 diluted overnight cultures (final inoculum size 10^6^ CFU/mL). After that, the plates were incubated with shaking at 120 rpm for 24 h in 37 °C. The MIC was calculated according to CLSI guidelines [20], as the lowest ESFE concentration causing 100% inhibition of the test organisms compared to the positive and negative control. The cell density was measured using a microplate reader at 620 nm (Tecan Elx800, Fitchburg, WI, USA). After incubation, the culture suspension was diluted and inoculated on tryptic soy agar (TSA) plates, incubated overnight at 37 °C and then the colony-forming units (CFUs) were counted. The MBC was determined as the lowest concentration of ESFE required to kill all bacterial cells [22].

### 2.5. Time–Kill Curves 

Time–kill curves were used to monitor bacterial growth and death over a gradient of ESFE concentrations versus time [22]. To assess the antimicrobial potential of ESFE on *S. aureus*, *P. aeruginosa*, *Bacillus subtilis*, *Escherichia coli* and *C. albicans,* the bacterial suspension (1 × 10^6^ CFU/mL) was mixed with the tested sample at concentrations of 0, 1/4, 1/2, 1 and 2 MIC and then incubated at 37 °C at 150 rpm. O.D. was determined spectrophotometrically at 620 nm every 2 h during the first 12 h and then after 24 h. 

### 2.6. Biofilm Inhibition Assay and MBIC

To evaluate the inhibitory potential of ESFE on bacterial and yeast biofilm formation, the MTP method was used against the *S. aureus* clinical isolate, *P. aeruginosa* and *C. albicans* [21,23]. Briefly, gradient concentrations (0.5–0.05, 2.5–0.3 and 6.0–0.7 mg/mL for *S. aureus*, *P. aeruginosa* and C. *albicans*, respectively) of ESFE was distributed into a flat-bottomed MTP with a tryptic soy broth media (TSB) supplemented with glucose (1%). An overnight culture of the test organisms was 1:100 diluted in TSB to an inoculum size of 1 × 10^5^ CFU/mL and loaded onto MTP and incubated for 48 h at 37 °C. O.D. was measured at 620 nm after the incubation period prior to the transfer of planktonic cells from the plates. The well content was then transferred without troubling the formed biofilms and the MTP wells were washed 3 times with phosphate-buffered saline (PBS) at pH 7.4 to remove the residue of the floated unbounded cells. The formed biofilm was then fixed with 200 μL methanol (95%) for 10 min in all wells. Crystal violet (0.3% *w*/*v*) was then added to each well using a multi-channel micropipette (CAPP, Berlin, Germany) and the plates were incubated at room temperature for 15 min. After that, the excess of crystal violet stain was removed, and the plates were washed with distilled sterile water. Finally, the crystal violet bound with biofilm was examined at this point and then photographed by an inverted microscope (Olympus Ck40, Tokyo, Japan) at 150×. For the quantitative determination of biofilm formation, 30% acetic acid was added to the wells and the color absorbance was measured at O.D_540nm_ by an automated microplate reader (Tecan Elx800, USA). The treated and the untreated wells were compared. For the estimation of the minimum biofilm inhibitory concentration (MBIC), an overnight culture of the test organism was adjusted to achieve an inoculum size of a 0.5 McFarland standard (1.5 × 10^8^ CFU/mL), and 150 μL of the media inoculated with the test organism was added to the wells and then incubated at 37 °C for 24 h. After incubation, the plates were washed 3 times with PBS (pH 7.4) to remove the planktonic unbounded bacteria and left to dry under aseptic conditions in an inverted position. Then 100 µL of the gradient dilutions of the ESFE in TSB media were transferred into the dried wells with the perfectly performing biofilms. The MTP were incubated at 37 °C for 20 h. For the determination of minimum biofilm inhibitory concentrations, all contents of the 96-well plates were cultured on TSA plates prior to washing with PBS and staining with crystal violet. The TSA plates were then incubated at 37 °C for 24 h. The MBIC value was considered as the concentration at which there was no growth of bacterial cells. [24].

### 2.7. HPLC-PDA-ESI-MS/MS 

The ESFE extract was analyzed utilizing a ThermoFinnigan LCQ-Duo ion trap mass spectrometer (ThermoElectron Corporation, Waltham, MA, USA) coupled with an ESI source (ThermoQuest Corporation, Austin, TX, USA) [25]. A ThermoFinnigan HPLC system using a Discovery HS F5 column (15 cm × 4.6 mm ID, 5 µm particles, Sigma-Aldrich Co Steinheim, Germany) was used. Water and acetonitrile (ACN) (Sigma-Aldrich GmbH, Berlin, Germany) (0.1% formic acid each) were used as a mobile phase adopting the same method reported in [26,27]. The autosampler surveyor ThermoQuest was used to inject the sample and Xcalibur software (Xcalibur^TM^ 2.0.7, Thermo Fischer Scientific, Waltham, MA, USA) was used to control the system. The MS operated in the negative mode [26,27]. 

### 2.8. Statistical Analyses

Three replicates were performed for each assay, and all resulted values were the averages of three independent experiments. To analyze the differences between a sample and the corresponding control, Student’s *t*-test was used. Differences were considered significant if the *p* values were <0.05.

## 3. Results

### 3.1. Antibacterial Activities

#### 3.1.1. Anti-Microbial, MIC and MBC of ESFE

ESFE displayed broad spectrum antimicrobial potential against Gram-positive and Gram-negative bacteria in addition to the yeast, *C. albicans*. ESFE growth inhibitory potential, reflected by the diameter of the inhibition zone, against Gram-positive *S. aureus* and *B. subtilis* bacteria was superior to its inhibitory potential against Gram-negative *E. coli* and *P. aeruginosa* (Table 1).

**Figure 1:**
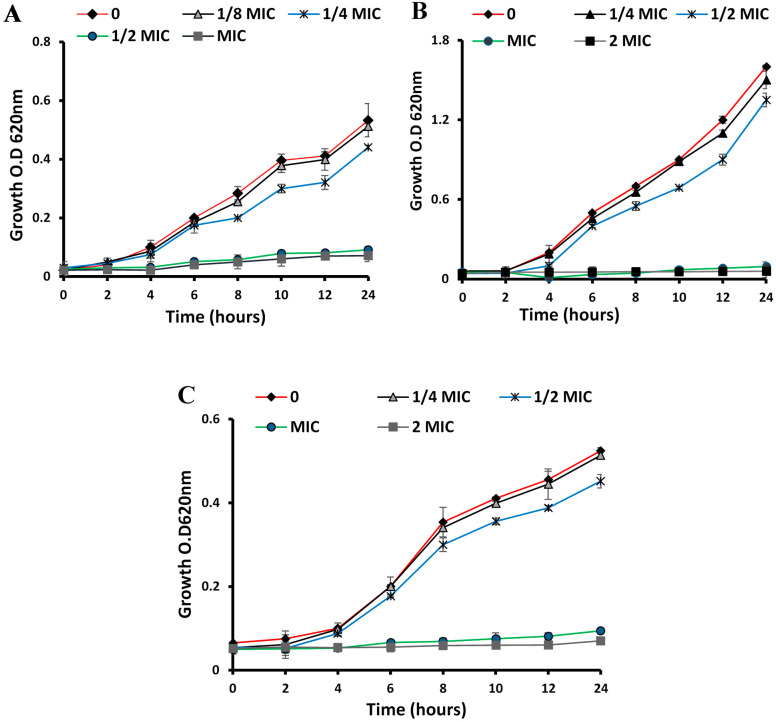
Time–kill curves illustrating the effect of different concentrations of *E. sideroxylon* flower extract on the growth of (**A**) *S. aureus* ACL51 (MRSA), (**B**) *P. aeruginosa* ATCC 27853, and (**C**) *C. albicans* ATCC 90028 every 2 h during the first 12 h and then after 24 h.

The inhibition zone reached 20 mm in diameter in the case of Gram-positive species at a low concentration of ESFE. There was no significant difference between the ESFE inhibitory potential against MRSA and MSSA. On the other hand, Gram-negative bacteria required a higher concentration of ESFE (up to 2.5 mg/mL) to obtain an inhibitory effect (Table 1). Moreover, a higher concentration of ESFE (5 mg/mL) was required to inhibit the growth of *C. albicans* with an inhibition zone diameter of 9 mm. MICs were determined against the previous organisms. The lowest MIC value (0.5 mg/mL) was recorded against MRSA and MSSA while the highest MIC value (3 mg/mL) was recorded against *C. albicans*. The MIC and MBC values against the Gram-positive, Gram-negative and yeast strains are listed in Table 2. The time–kill curve showed that a 1/4 MIC of ESFE had no growth inhibitory potential against the three biofilm-forming organisms: *S. aureus* ACL51 (MRSA), *P. aeruginosa* and *C. albicans* (Figure 1). Thus, this concentration was selected for the antibiofilm assay.

#### 3.1.2. Biofilm Inhibition Activity

**Figure 2:**
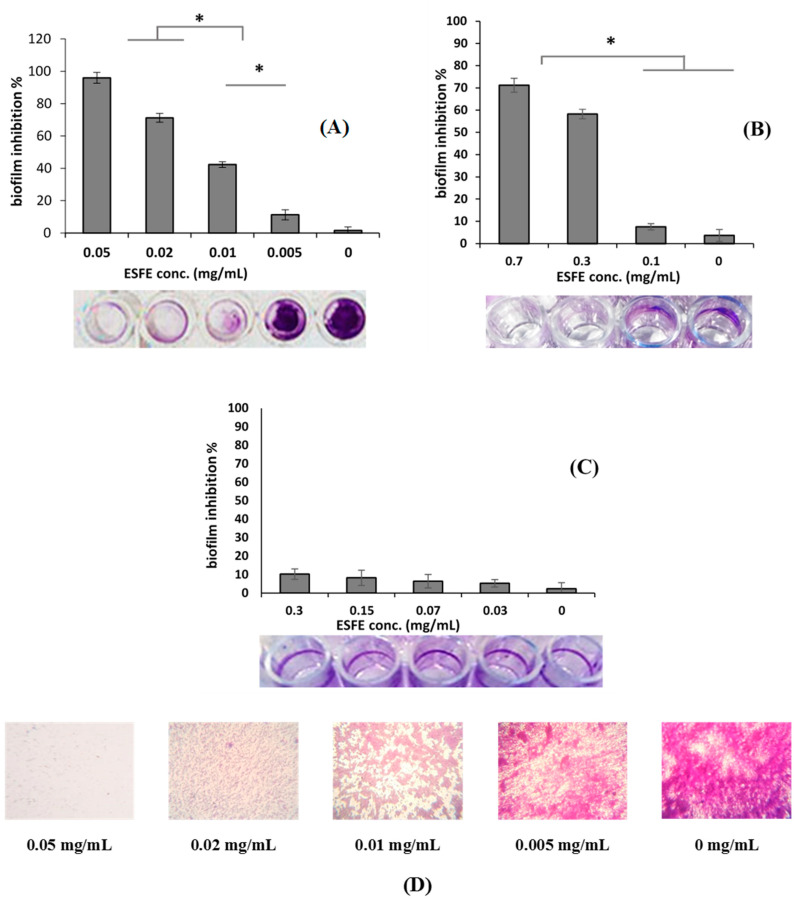
Biofilm inhibition percentage of *E. sideroxylon* flower extract (ESFE) against the biofilm formation of (**A**) *S. aureus* ACL51 (MRSA), (**B**) *C. albicans* ATCC 90028, and (**C**) *P. aeruginosa* ATCC 27853. (**D**) Microscopic images (×150) illustrate the effect of different sub-MICs of ESFE on the biofilm formation of the highly producing biofilm *S. aureus* ACL51 (MRSA). Significant differences are indicated by * *p* < 0.05.

ESFE demonstrated robust biofilm inhibitory activities against MRSA and *C. albicans* at sublethal concentrations (*p* < 0.05; Figure 2). ESFE exhibited significant dose-dependent inhibition of MRSA biofilm formation up to 95.9% (*p* < 0.05) at a concentration of 0.05 mg/mL. At a concentration of 0.7 mg/mL, the ability of ESFE to eradicate *C. albicans* biofilm formation reached 71.2% (*p* < 0.05) in a dose-dependent manner. On the other hand, the formation of *P. aeruginosa* biofilm was not affected by the sub-lethal dose of ESFE (0.3–0.03 mg/mL). The MBIC against MRSA and *C. albicans* was 0.01 and 0.3 mg/mL, respectively.

### 3.2. Metabolic Profiling

**Figure 3:**
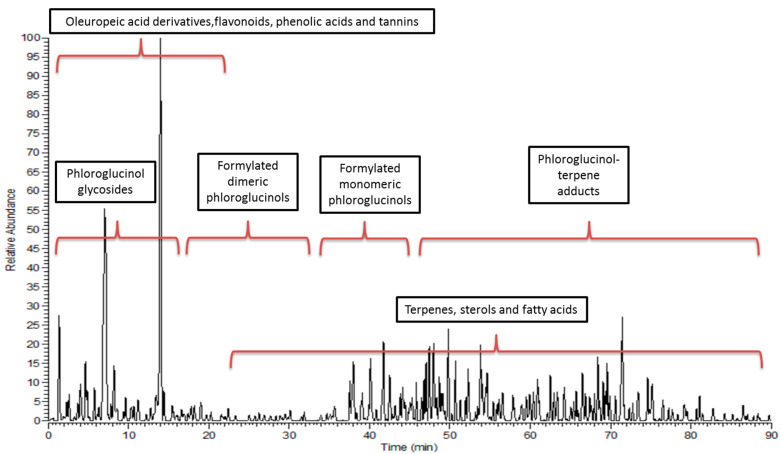
LC-MS/MS profile of the methanolic extract of *E. sideroxylon* flowers.

The HPLC-PDA-ESI-MS/MS analysis of the ESFE revealed the tentative identification of 83 compounds: 21 phloroglucinol, 18 terpenes, 16 flavonoids, 7 oleuropeic acid derivatives, 7 ellagic acid derivatives, 6 gallic acid derivatives, 3 phenolic acids, 3 fatty acids and 2 miscellaneous. The observed molecular weights, retention times (rt), fragment ions and chemical class of each compound, and their identities, are presented in Table 3. Figure 3 represents the LC-MS profile of the extract.

The identified metabolites are a complex mixture of several formylated phloroglucinols (FPs), polyphenolics (Hydrolyzable tannins, phenolic acids, flavanone glycoside, and oleuropeic acid derivatives) and terpenoids. The peaks’ identities were predicted using an in-house metabolite database, according to the parent masses and retention times and based on the comparison of their mass spectra with the reported data from the genus *Eucalyptus*, as well as the Plant Dictionary MS Database and METLIN.

**Phloroglucinols:** Twenty-one phloroglucinols were identified. They included three formylated monomeric phloroglucinols, five formylated dimeric phloroglucinols, four phloroglucinol glycosides and nine phloroglucinol-terpene adducts (phloroglucinol meroterpenoids). Compound (6) was tentatively identified as sideroxylonal A/B/C. Its molecular ion peak [M^−^H]^−^ appeared at *m/z* 499. Upon fragmentation, it gave an ion at *m/z* 471 and at *m/z* 453 due to loss of CO (28 dalton) and subsequent loss of the H_2_O moiety (18 dalton). Additionally, a daughter ion at *m/z* 249 corresponding to the isopentyl diformyl phloroglucinol moiety was also detected. These isomers were eluted at retention times of 4.66, 8.41 and 10.96 min, which show that several isomers of sideroxylonal were present in ESFE. This comes in agreement with the findings of Moore et al. [45], who reported that macrocarpals and sideroxylonals are the most common groups of FPs reported in the genus *Eucalyptus*. 

**Figure 4:**
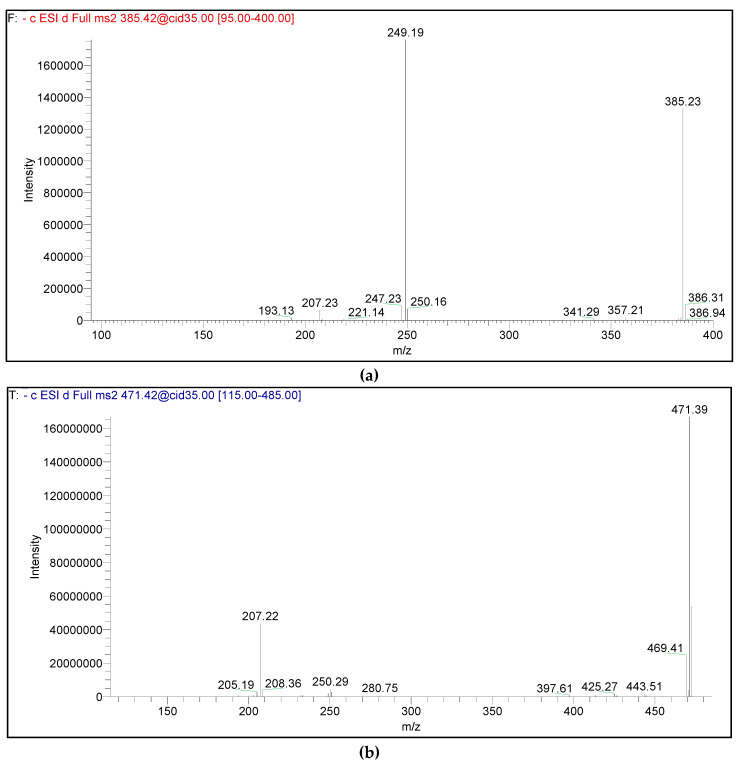
MS-MS product ions of [M−H]^−^ ions at (**a**) *m/z* 385 of monoterpene hydrocarbons euglobals (**b**) *m/z* 471 of sesquiterpene alcohols macrocarpals.

Phloroglucinol-terpene adducts (Euglobals and macrocarpals) were also detected. The identified euglobal (21) was detected at *m/z* 385. It was characterized by an intense fragment daughter ion at *m/z* 249 and a less intense ion at *m/z* 207 (Figure 4a) [46]. On the other hand, the tentatively identified macrocarpals (13, 14, 17 and 20) are sesquiterpene adducts with molecular ions detected at *m/z* 489, 471 and 453, respectively. Upon fragmentation, they yielded an intense product ion at *m/z* 207, with a weaker product ion at *m/z* 249 (Figure 4b) [15,46]. The fragment produced at *m/z* 249 in macrocarpals corresponds to the isopentyl diformyl phloroglucinol moiety that resulted from the typical cleavage of the sesquiterpene moiety.

Phloroglucinol glycosides were found as well. Myrciaphenone B (9), a phloroglucinol glycoside attached to the galloyl moiety, and thus the 169 dalton daughter ion corresponding to the galloyl moiety, was detected. Another daughter ion at *m/z* 331 due to the loss of the galloyl and hexose moieties [M−H-169-162]^−^ was also observed. The other three tentatively identified phloroglucinol glycosides were eucalmainoside A, B and C. The molecular ion of eucalmainoside A (10) was detected at 301 corresponding to 2-methylphloroglucinol-*O-β*-D-glucopyranoside. The molecular ions of compounds (12 and 11) at *m/z* 315 and 329 exceeded that of eucalmainoside A (10) by 14 (CH_3_ moiety) and 28 (CHO moiety) dalton, respectively, corresponding to 2,4-dimethylphloroglucinol- *O*-*β*-D-glucopyranoside (eucalmainoside B) and 2,4,6-trihydroxy- 3-methylbenzaldehyde-2-*O*-*β*-D-glucopyranoside (eucalmainoside C), respectively.

**Oleuropeic acid derivatives**: Seven oleuropeic acid derivatives were tentatively identified (22–28). All of them are oleuropeic acid-containing carbohydrates (oleuropeic acid glucosides); i.e., carbohydrate monoterpene esters. There are two types of oleuropeic acid glucosides characterized in the studied extract. The first type contains a terpene moiety other than oleuropeic acid and is represented by globulusin A (23). It is a β-glucopyranose ester of gallic acid and 2-hydroxy-1,8-cineol. The second type contains a polyphenolic moiety. This type is represented by the cypellocarpines (24,25 and 28); a group of oleuropeic acid glucosides that differs only in the phenolic moiety incorporated in their structures. Globulusin A/B (23 and 27) are monoterpene glycosides conjugated with gallic acid. Globulusin A (23) fragmentation showed peaks at *m/z* 313 [M − H-galloyl]^−^, 169 [M-hydroxyl cineole-162]^−^ and 151 [M − H-162-169]^−^. Daughter ions at *m/z* 313 (oleuropeic acid methyl ester), 169 (galloyl moiety), 151 [M − H-162-169]^−^ and 183 (oleuropeic acid methyl ester) were detected due to fragmentation of globulusin B (27). The fragmentation pattern of globulusin A and globulusin B is in accordance with what was previously reported in [34]. The MS spectrum of Compound (26) showed a prominent fragment (*m/z* at 463) corresponding to the loss of the oleuropeic acid moiety of eucalmaidin D or cypellogin A/B. Compound (26) fragmentation will be discussed under the flavonoids section. 

**Flavonoids and their glycosides**: The sixteen characterized flavonoids are 11 flavonols (29–31,33,34,36,38–42), 4 flavones (35,37,43–44) and a flavanone (32). The predominating class was the flavonols. They were characterized, in the spectra of their deprotonated glycosides, by ions corresponding to the deprotonated aglycones at *m/z* 285, 301 and 315 (for kaempferol, quercetin and isorhamnetin, respectively), generated by the loss of the sugar units. Furthermore, fragment ions at *m/z* 255, 271 and 285 were detected corresponding to [M-H-CO-H]^−^, resulting from fragmentation of the kaempferol, quercetin and isorhamnetin aglycones, respectively [47]. A total of 16 flavonoids were tentatively identified, of which eleven were *O*-glycosides, one was *C*-glycoside (37) and four were flavonoid aglycones (41–44); this in addition to eucalmaidin D, quercetin-4’-*O*-(6-*O*-oleuropeoyl)-*R*-D-glucopyranoside (26), which was listed under the abovementioned oleuropeic acid derivatives class. Eucalmaidin D (26) is a rare example of an α-configured glucoside found in nature. It was detected before in the leaves of the same plant [15] and was isolated from the leaves of *E. maideni* as well [48]. The nature of the sugars in *O*-glycosides could be revealed from the elimination of the sugar residue. The primary sugar can be identified as hexose, pentose, and deoxyhexose if the intermediate ion appears at 162, 132, and 146 dalton from molecular ions, respectively [49]. 

**Phenolic acids**: Three phenolic acids (45–47) were tentatively identified in ESFE, namely, gallic, chlorogenic/neochlorogenic and ferulic acid. Their fragmentation pattern is in agreement with that reported for phenolic acids in [50], where all of them were previously reported from the genus *Eucalyptus* [37].

**Gallic acid derivatives**: Results revealed the presence of six gallic acid derivatives, three of which are gallic acid glycosides (48, 49 and 52), in addition to myrciaphenone B (9), globulusin A (23) and globulusin B (27) discussed before under the phloroglucinol glycosides and oleuropeic acid derivatives sections. Product ions due to the loss of the galloyl moieties [M − H-169]^−^ and galloyl moiety (169 dalton) were observed in their mass spectra (Table 3). 

**Ellagic acid derivatives**: Compound (54) was assigned to ellagic acid. It was characterized by [M-H]^−^ at *m/z* 301. Its MS/MS fragmentation was typical to the reported ellagic acid pattern [16]. The molecular ions of Compounds (58–60) exceeded that of ellagic acid by 14, 28 and 42 dalton, corresponding to the extra methyl groups (1-CH_3_, 2-CH_3_ and 3-CH_3_). Their fragmentation was characterized by a loss of 15 dalton [M-CH_3_-H]^−^ due to the loss of the methyl radical. They were tentatively identified as methylellagic, dimethylellagic, and trimethylellagic acids. 

**Triterpenes and sterols**: About 18 triterpenoids were characterized: oleananes (75 and 76), ursanes (62,64–68, and 69) and lupanes (63), representing the classes of pentacyclic triterpenes. Compound (62) was assigned to asiatic acid lactone ((2a,3b)-2,3,23-trihydroxy-13,28-epoxyurs-11-en-28-one). This is the third report of the presence of this pentacyclic triterpenoid with an 11-en-28,13*β*-olide structure in nature after being detected in *E. camaldulensis* [51] and *E. sidroxylon* leaves [15]. Four pentacyclic triterpenes with an *O*-*p* coumaroyl substitution were detected. The observed daughter ions at *m/z* 455, 485, 437 and 471 in the MS spectra of (69,70,73 and 74), respectively, were due to the loss of 162 dalton; corresponding to losing the *O*-*p* coumaroyl group at C_3_. The [M-H]^−^ of Compounds (70) and (74) (647 and 633, respectively) exceeded that of *O*-*p* coumaroyl maslinic/alphitolic acid (69) by 30 and 16 dalton, respectively. This indicates an extra OCH_3_ in Compound (70) and OH in Compound (74), which, in turn, confirms their identification as eucalyptic acid (Eucalyptolic) (70) and *O*-*p* coumaroyl tormentic acid (74). The observed daughter ion in Compound (70) at *m/z* 617 is due to the loss of the OCH_3_ group [M-H-OCH_3_]^−^.

Fatty acids: Three oxygenated fatty acids (saturated and unsaturated) were annotated. Their fragmentation was in agreement with that of the hydroxylated fatty acids [52,53]. Compounds (79) and (81) with molecular ion peaks at *m/z* 329 and *m/z* 295 were assigned to trihydroxy octadecenoic and hydroxy octadecadienoic. The mass difference of 2 × 16 dalton between Compounds (79) and (81) suggested an extra two hydroxyl groups in addition to a 2 dalton difference corresponding to the saturation of one of the double bonds of (81). Our identification was confirmed by the appearance of a product ion at *m/z* 277 [M-H-H_2_O]^−^ corresponding to the loss of a water molecule.

Miscellaneous compounds: Vomifoliol (82), a monoterpene derivative related to abscisic acid, and withanolide A (83) were tentatively identified. Withanolide is a naturally occurring C-28 steroidal lactone with an ergostane-type skeleton.

## 4. Discussion

The inhibition of biofilm is considered an important way for the treatment of bacterial infections [8]. Searching for bacterial virulence factors inhibitors is very important due to the well-known ability of pathogens to develop different mechanisms of antibiotics resistance. Exploring phyto-constituents seems necessary to avoid synthetic drugs’ toxic side effects [54]. Results showed that ESFE had broad spectrum antibacterial activity against Gram-positive and Gram-negative bacteria and yeast. The Gram-positive bacteria *S. aureus* and *B. subtilis* bacteria were more susceptible than the Gram-negative bacteria *E. coli* and *P. aeruginosa* to the inhibitory activity of ESFE, while *C. albicans* showed the smallest inhibition zone. Furthermore, at sub-lethal concentrations, ESFE showed significant anti-biofilm activity against MRSA and *C. albicans* (95.9 and 71.2% inhibition, respectively) in a dose-dependent manner. 

The observed higher potency of ESFE towards Gram-positive bacteria than Gram-negative bacteria is in accordance with that reported for three *Eucalyptus* species (*E. globulus*, *E. radiate* and *E. citriodora)* against different Gram-positive (VRE and MRSA) and Gram-negative (*P. aeruginosa, E. coli, Klebsiella pneumonia* and *Acineto*) bacteria [55]. The tested sample was active with variable degrees against Gram-positive bacteria with regards to their chemical constituents. However, the tested samples were almost inactive against multidrug-resistant Gram-negative bacteria due to the differences in cell wall sub-structures. In the same stream, *E. globulus* and *E. camaldulensis* inhibitory activity was evaluated by micro-atmosphere, aromatogramme and germs in suspension assays against *E. coli* and *S. aureus.* They demonstrated an inhibitory effect on both bacteria, but to a lesser extent on *E. coli* [56]. The observed anti-*Candida* potential of ESFE matched that reported about the significant activity of *E. globulus* and *E. citriodora* against several *Candida* species [57]. 

HPLC-PDA-ESI-MS/MS results revealed that ESFE is rich in phloroglucinols (twenty-one secondary metabolites) and flavonoids (sixteen secondary metabolites) in addition to several phenolic acid derivatives. Consequently, the obtained potent antimicrobial potency might be due to the intrinsic acidic characters of this phenolic metabolites, which create a lethal antibacterial environment [58].

The anti-biofilm activity of phloroglucinols and flavonoids is well documented [7,59,60]. Their phenolic nature could be responsible for such activity. Many mechanisms were suggested for the antibiofilm activity of plant phenolics, including modulation of bacterial cells communication, interference with motility, surface hydrophobicity and charge and downregulation of the genes responsible for the biofilm formation [59,61,62].

The most common acylpholoroglucinols in *Eucalyptus* are the diformyl monomeric phloroglucinols, phloroglucinol glycosides, dimeric acylphloroglucinols, phloroglucinol-sesquiterpene and acylphloroglucinol-monoterpene adducts [31]. All these classes were detected in the *E. sideroxylon* flowers. It was found to be rich in sideroxylonals, macrocarpals and euglobals.

The antibacterial activity of the pholoroglucinol, eucalyptin A, was previously evaluated against *S. epidermidis*, *S. aureus* and *P. aeruginosa*. It showed potent antimicrobial activity against the two biofilm-producing strains *S. epidermidis* and *S. aureus*, with MIC values of 1.7 and 3.5 mg/mL, respectively, but was inactive against the Gram-negative *P. aeruginosa* [63].

The desmethyl eucalyptin detected in ESFE was proven to exhibit bacteriostatic activity against methicillin-resistant and sensitive *S. aureus* strains (MRSA and MSSA). It also exhibited potent antibiofilm potential at sub-MICs and inhibited staphyloxanthin biosynthesis and decreased the survival rates of MRSA (73.1%) and MSSA (54.6%). Disintegration of the outer membrane, irregular shape of the cells and leakage of cytoplasm were also observed after treatment of MSSA with desmethyl eucalyptin and its methylated derivative, eucalyptin [7].

The monoterpene acid glucose ester, eucaglobulin, was previously isolated from *E. globulus* leaves [34,64,65] and fruits [66] and was reported to exert significant inhibition against *C. albicans, E. coli* and *S. aureus* [65]. The flower extract of *E.* *sideroxylon* is a rich source of hydrolyzable tannins (6 gallic and 7 ellagic acid derivatives). This is in accordance with two recent studies that reported the presence of several hydrolysable tannins in the bark of *E.* *sideroxylon* [16] and determined their quantity [67]. The *S. aureus* biofilm inhibitory activity of gallic [68] and ellagic acids [69] at subinhibitory concentrations was also reported. It was reported that ellagic acid/ellagic acid derivatives could limit biofilm formation of *S. aureus* to an extent that could be correlated with the increased antibiotic susceptibility [70]. Gallic and ellagic acids also inhibited biofilm formation of *E. coli* [71,72,73].

The potent antibiofilm activity of ESFE against MRSA and *C. albicans* might be attributed to the synergistic effects of all secondary metabolites identified by HPLC-PDA-ESI-MS/MS, including phloroglucinols, OAG, flavonoids and tannins. It is worthy to mention that Myrtaceae phloroglucinols may act as biofilm inhibitors more effectively in their pure state rather that in the crude extract [7]. Chemical synthesis studies have attempted to produce phloroglucinols [28]; however, this was a difficult and costly process. Consequently, it is highly recommended to prepare *E. sideroxylon* flower phloroglucinol-rich extracts or to isolate and structurally elucidate its phloroglucinols, to be studied as a nucleus for exploring novel anti-biofilm agents.

## 5. Conclusions

This study highlights the promising role of *E. sideroxylon* flowers as an anti-biofilm agent against both Gram-positive bacteria and yeast. The HPLC-PDA-MS/MS fingerprint of the *E. sideroxylon* flowers represents the first complete secondary metabolites profile of the plant. The efficacy of the extract against MRSA and *C. albicans* could prove greater efficacy after further in vivo and clinical studies. The combination of *E. sideroxylon* flower extract with other natural antibiofilm agents could reduce the risk of bacterial and yeast strain resistance to various synthetic drugs. 

## Figures and Tables

**Table 1:** Antimicrobial potential of ESFE.

Strain		ESFE (mg/mL)							Gentamicin (10 µg/mL)
		20	10	5	2.5	1.25	0.6	0.12	
Gram positive	MSSA	+++	+++	+++	+++	++	+	−	+++
	MRSA	+++	+++	+++	+++	++	+	−	+++
	*B. subtilis*	+++	+++	+++	++	++	+	−	+++
Gram negative	*E. coli*	+++	++	++	+	−	−	−	+++
	*P. aeruginosa*	+++	+++	++	++	−	−	−	+++
Yeast	*C. albicans*	++	++	+	−	−	−	−	++

ESFE: *E. sideroxylon* flower extract; MRSA: methicillin-resistant *Staphylococcus aureus*; MSSA: methicillin-sensitive *S. aureus*; Zone of inhibition −: < 5 mm, +: 5–9 mm, ++: 10–19 mm, +++: > 20 mm.

**Table 2:** MIC and MBC of ESFE against the tested organisms.

Strain		ESFE (mg/mL)	
		MIC	MBC
Gram positive	MSSA	0.5	1.0
	MRSA	0.5	1.0
	*B. subtilis*	1.2	2.5
Gram negative	*E. coli*	1.2	2.5
	*P. aeruginosa*	1.2	2.5
Yeast	*C. albicans*	3	6

ESFE: *Eucalyptus sideroxylon* flower extract; MBC: minimum bactericidal concentration; MIC: minimum inhibitory concentration, MRSA: methicillin-resistant *Staphylococcus aureus*; MSSA: methicillin-sensitive *S. aureus*.

**Table 3:** Secondary metabolites identified in the methanolic extract of *E. sideroxylon* flowers.

No.	Identification	R_t_ (min)	[M−H]^−^	Main Fragments	Ref.
**Phloroglucinol**					
Formylated monomeric phloroglucinols					
1	Jensenone	27.98	265	249, 193, 165, 149	
2	Grandinol	37.98	251	236, 167	[28]
3	Homograndinol	40.73	265	250, 207	
Formylated dimeric phloroglucinols					
4	Dehydro-eucalyptusdimer C	7.72, 10.88	725	563, 441, 423, 361, 207	[29]
5	Eucalyptusdimer A/B	10.28	713	609, 503, 489, 457, 207	[29]
6	Sideroxylonal A/B/C	4.66, 8.41, 10.96	499	471, 453, 423, 207, 165	[15]
7	Loxophlebal A	8.97	471	281, 249, 207	[28]
8	Eucalyprobusone A	27.96	459	319, 251, 249, 209, 181	[29]
Phloroglucinol glycosides					
9	Myrciaphenone B	1.47	481	331, 319, 301, 183, 163	[30]
10	Eucalmainoside A	6.30	301	257, 229, 183, 177, 169	[31]
11	Eucalmainoside C/Myrciaphenone A	15.35	329	229, 183, 171, 169, 167	[32]
12	Eucalmainoside B	19.61	315	301, 249, 183, 169, 151	[31]
Phloroglucinol-terpene adducts (phloroglucinol meroterpenoids)					
13	Macrocarpal E/Eucalyptone/Eucalyptals B/E	44.71, 63.04	485	471, 409, 439, 373, 207	[15]
14	Macrocarpal J/I	39.69	489	471, 324, 249, 207	
15	Eucalrobusone R/O	35.84	469	423, 249, 207	
16	(Iso)leptospermone	43.56	265	250, 207, 112	
17	Macrocarpal A/B/D/K/H/L-Eucalyptin A/B	45.30, 47.87	471	469, 453, 385, 249, 207	
18	Eucalyptal A/C/Eucalrobusone D	49.55	467	453, 249, 207	
19	Eucalyptone G	58.44	675	453, 397, 250, 207	[9]
20	Macrocarpal C/G	61.95, 62.99	453	428, 407, 250, 207, 165	[33]
21	Euglobals G1-G12/R	63.39, 63.79	385	249, 207	[15]
**Oleuropeic acid derivatives**					
22	Galloylglucose	2.01	331	331, 313, 169	[34]
23	Globulusin A	4.08	483	313, 353, 183, 169, 151	
24	Cypellocarpin B	22.66	537	453, 385, 209, 183, 191	[35]
25	Cypellocarpin C (Camaldulenside)	24.67	519	353, 335, 245, 205, 183	
26	Eucalmaidin D/Cypellogin A/B	17.78, 23.54	629	519, 469, 463, 301, 183	[15]
27	Globulusin B/Eucaglobulin/Cypellocarpin A	28.27	497	437, 331, 313, 183, 169	[34]
28	Dihydrocypellocarpine C	29.10	521	489,441, 353, 279, 160	[35]
**Flavonoids and flavonoid glycosides**					
29	Quercetin *O*-sophoroside	7.97	625	463, 301, 271, 151	[36]
30	Quercetin rutinoside (Rutin)	10.28	609	463, 301, 271	[31]
31	Quercetin *O*-arabinopyranoside-gallate	11.92	585	301, 269	[37]
32	Hydroxytetramethoxy-flavone-*O*-glucopyranoside	12.05	519	447, 353, 335, 205	[15]
33	Isorhamnetin *O*-rutinoside (Narcissin)	12.50	623	315, 300, 285, 271, 255	[37]
34	Quercetin *O*-glucopyranoside-gallate	13.55	615	301, 271	[38]
35	Luteolin *O*-rutinoside (Scolymoside)	16.80	593	429, 285	[37]
36	Quercetin *O*-arabinofuranoside/Quercetin *O*-arabinopyranoside	19.61, 24.67	433	301, 271	
37	Homoorientin (Isoorientin)	20.95	447	315	
38	Quercetin *O*-glucopyranoside	22.24	463	301, 271, 151	
39	Quercetin *O*-rhamnoside	22.34	447	301, 271	
40	Kaempherol *O*-glucopyranoside/Luteolin *O*- glucopyranoside	25.59	447	285, 255	
41	Trimethoxykaempferol	26.53	327	309, 283, 255	[9]
42	Isorhamnetin	27.55	315	300, 285, 151, 107	[37]
43	Desmethyl eucalyptin	31.92	311	297, 293, 267, 249	[39]
44	Sideroxylin	38.17	311	296, 249, 207	[40]
**Phenolic acids**					
45	Gallic acid	2.01	169	169, 125	[28]
46	Chlorogenic/Neochlorogenic acid	2.21 2.25	353	233, 191	[37]
47	Ferulic acid	28.13	193	165	
**Gallic acid derivatives**					
48	*O*-galloyl-*O*-HHDP-glucose	1.54	633	463, 301, 275, 169	[41]
49	Tri-*O*-galloylglucose	2.45	635	483, 477, 465, 169	[41]
50	Epicatechin gallate	3.21	441	271, 169	[37]
51	Tellimagradin I	3.35	785	634, 617, 301, 169	[28]
52	Coumaroyl-digalloylhexoside	5.07	629	463, 459, 313, 169	[42]
53	Sinapaldehyde	14.32	207	179, 161	[28]
**Ellagic acid derivatives**					
54	Ellagic acid	2.17	301	273, 257, 229	[37]
55	Methylellagic acid acetyl hexoside	4.53	503	373, 315, 313, 183	[43]
56	Ellagic acid deoxyhexoside	5.00	447	315, 301, 261, 185	[16]
57	Dimethylellagic acid hexoside	1.51	475	327, 301	[28]
58	Methylellagic acid	5.86	315	300, 269, 180	[16]
59	Dimethyl ellagic acid	11.42	329	315, 163	
60	Trimethyl ellagic acid	11.74	343	328, 315, 249	
**Terpenes**					
61	Hydroxy-*O*-acetylhydroshengmanol-*O*-xylopyranoside	38.11	695	649, 533, 520, 225	[15]
62	Asiatic acid lactone	39.88	485	403, 433, 251, 207	
63	Betulin	40.14, 42.48, 49	443	443, 399, 165	
64	Hydroxy ursolic/betulinic acid	45.30, 47.93, 59.77	471	453, 427, 380	
65	Euscaphic/asiatic/arjunolic acid	45.56	487	469, 453, 423, 207	
66	Trihydroxy-oxoursenoic acid	46.63	473	454, 375, 311	
67	Nor-ursene-diol	47.20	427	301, 297, 207	
68	Acetyl ursolic/Acetyl oleanolic acid/Acetobetulinic acid	48.33	497	485, 249, 207	
69	*O*-Coumaroyl maslinic/Alhitolic acid	48.40, 48.53, 49.22	617	574, 471, 455, 453, 249	
70	Eucalyptic (eucalyptolic) acid	49.27	647	632, 617, 497, 485, 397	
71	Lupeol acetate	57.17	467	439, 249, 209	[44]
72	Eucalyptanoic acid	57.62	453	249, 207	[15]
73	Bryocoumaric acid	61.91	599	555, 469, 437, 385, 249	
74	*O*-coumaroyl tormentic acid	62.04	633	471, 453, 207	
75	Ursolic/Oleanolic/betulinic acid	62.78, 65.38	455	398, 251, 249, 207	
76	4-Methoxycinnamoyloleanolic acid methyl ester	63.51	629	614, 585, 485, 249	
77	Ursolic acid lactone	63.55	453	325	
78	Nor triterpene	64.45	453	385, 249	
**Fatty acids**					
79	Trihydroxy octadecenoic acid	50.23, 67.72	329	311, 293, 275, 229	[15]
80	Hydroxy tetracosanoic acid	50.27, 57.44	383	363, 326, 309, 272	
81	Hydroxy octadecadienoic acid	82.44	295	277, 171	
**Miscellaneous**					
82	Vomifoliol	25.61	223	208, 139	[28]
83	Withanolide A	65.63	469	425, 249, 205	[15]

## References

[1] Khan F., Pham D.T.N., Oloketuyi S.F., Manivasagan P., Oh J., Kim Y.-M. (2020). Chitosan and Their Derivatives: Antibiofilm Drugs Against Pathogenic Bacteria. Colloids Surf. B Biointerfaces.

[2] Desouky S.E., El-Gamal M.S., Mohammed A.F., Abu-Elghait M.A. (2014). Determination of Some Virulence Factors in Staphylococus Spp. Isolated from Clinical Samples of Different Egyptian Patients. World Appl. Sci. J..

[3] Otto M. (2019). Staphylococcal Biofilms. Microbiol. Spectr..

[4] Kaur S., Sharma N., Aanchal A.G., Sharma A., Sharma A., Sharma V. (2018). Anti-biofilm Potential of Aqueous Eucalyptus Leaf Extract against Nosocomial Pathogens: Staphylococcus and Pseudomonas Aeruginosa. Pharm. Innov. J..

[5] Lu L., Hu W., Tian Z., Yuan D., Yi G., Zhou Y., Cheng Q., Zhu J., Li M. (2019). Developing Natural Products as Potential Anti-Biofilm Agents. Chin. Med..

[6] Barriuso J. (2015). Quorum Sensing Mechanisms in Fungi. AIMS Microbiol..

[7] Shehabeldine A.M., Ashour R.M., Okba M.M., Saber F.R. (2020). Callistemon Citrinus Bioactive Metabolites as New Inhibitors of Methicillin-Resistant Staphylococcus Aureus Biofilm Formation. J. Ethnopharmacol..

[8] Mostafa I., Abbas H.A., Ashour M.L., Yasri A., El-Shazly A.M., Wink M., Sobeh M. (2020). Polyphenols from Salix Tetrasperma Impair Virulence and Inhibit Quorum Sensing of Pseudomonas Aeruginosa. Molecules.

[9] Mohamed G.A., Ibrahim S.R. (2007). Eucalyptone G, a New Phloroglucinol Derivative and Other Constituents from Eucalyptus Globulus Labill. Arkivoc.

[10] Bhuyan D.J., Vuong Q.V., Chalmers A.C., van Altena I.A., Bowyer M.C., Scarlett C.J. (2017). Phytochemical, Antibacterial and Antifungal Properties of an Aqueous Extract of Eucalyptus Microcorys Leaves. S. Afr. J. Bot..

[11] Bailey L.H. (1958). Manual of Cultivated Plants.

[12] Umberto Quattrocchi F.L.S. (1999). World Dictionary of Plant Names.

[13] Ashour H.M. (2008). Antibacterial, Antifungal, and Anticancer Activities of Volatile Oils and Extracts from Stems, Leaves, and Flowers of Eucalyptus Sideroxylon and Eucalyptus Torquata. Cancer Biol. Ther..

[14] Elaissi A., Salah K.H., Mabrouk S., Larbi K.M., Chemli R., Harzallah-Skhiri F. (2011). Antibacterial Activity and Chemical Composition of 20 Eucalyptus Species’ Essential Oils. Food Chem..

[15] Okba M.M., El Gedaily R.A., Ashour R.M. (2017). UPLC–PDA–ESI–qTOF-MS Profiling and Potent Anti-HSV-II Activity of Eucalyptus Sideroxylon Leaves. J. Chromatogr. B.

[16] Ashour R.M., Okba M.M., Menze E.T., El Gedaily R.A. (2019). Eucalyptus Sideroxylon Bark Anti-inflammatory Potential, Its UPLC-PDA-ESI-qTOF-MS Profiling, and Isolation of a New Phloroglucinol. J. Chromatogr. Sci..

[17] Santos B.M.d., Zibrandtsen J.F., Gunbilig D., Sørensen M., Cozzi F., Boughton B.A., Heskes A.M., Neilson E.H.J. (2019). Quantification and Localization of Formylated Phloroglucinol Compounds (Fpcs) in Eucalyptus Species. Front. Plant Sci..

[18] Hamed A., Abdel-Razek A.S., Araby M., Abu-Elghait M., El-Hosari D.G., Frese M., Soliman H.S., Stammler H.G., Sewald N., Shaaban M. (2020). Meleagrin from Marine Fungus Emericella Dentata Nq45: Crystal Structure and Diverse Biological Activity Studies. Nat. Prod. Res..

[19] Stankov S., Fidan H., Stefanova G., Kostova I., Damyanova S., Dimitrova-Dyulgerova I., Ercisli S., Stoyanova A. (2020). Chemical Composition and Antimicrobial Activity of Essential Oil from Aerial Part (Leaves and Fruit) of Eucalyptus gomphocephala DC. J. Essent. Oil Plants.

[20] CLSI (2011). Performance Standards for Antimicrobial Susceptibility Testing.

[21] Abdelhameed R.M., Abu-Elghait M., El-Shahat M. (2020). Hybrid Three Mofs Composites (ZIF-67@ ZIF-8@ MIL-125-NH2): Enhancement the Biological and Visible-Light Photocatalytic Activity. J. Environ. Chem. Eng..

[22] Qian W., Liu M., Fu Y., Zhang J., Liu W., Li J., Li X., Li Y., Wang T. (2020). Antimicrobial Mechanism of Luteolin against Staphylococcus Aureus and Listeria Monocytogenes and Its Antibiofilm Properties. Microb. Pathog..

[23] Desouky S.E., Hassan S.E., El-gamal M.S., Ragab M.A.E.T.I., Emam M. (2017). Effect of Salvia Egypticae and Foeniculum Vulgara Extracts on Quorum Sensing and Biofilm Formation of Methicillin Resistant/Sensitive Staphylococcus Aureus Isolates. World J. Pharm. Med. Res..

[24] Černohorská L., Votava M. (2008). Antibiotic Synergy against Biofilm-Forming Pseudomonas Aeruginosa. Folia Microbiol..

[25] Sobeh M., Mahmoud M.F., Sabry O.M., Adel R., Dmirieh M., El-Shazly A.M., Wink M. (2017). HPLC-PDA-MS/MS Characterization of Bioactive Secondary Metabolites from Turraea Fischeri Bark Extract and Its Antioxidant and Hepatoprotective Activities in Vivo. Molecules.

[26] El-Hawary S.S., Mubarek M.M., Lotfy R.A., Hassan A.R., Sobeh M., Okba M.M. (2020). Validation of Antidiabetic Potential of Gymnocarpos decandrus Forssk. Nat. Prod. Res..

[27] El-Hawary S.S., Sobeh M., Badr W.K., Abdelfattah M.A., Ali Z.Y., El-Tantawy M.E., Rabeh M.A., Wink M. (2020). HPLC-PDA-MS/MS Profiling of Secondary Metabolites from Opuntia Ficus-Indica Cladode, Peel and Fruit Pulp Extracts and Their Antioxidant, Neuroprotective Effect in Rats with Aluminum Chloride Induced Neurotoxicity. Saudi J. Biol. Sci..

[28] Singh I.P., Sidana J., Bharate S.B., Foley W.J. (2010). Phloroglucinol Compounds of Natural Origin: Synthetic Aspects. Nat. Prod. Rep..

[29] Qin X.-J., Feng M.-Y., Liu H., Ni W., Rauwolf T., Porco J.A., Yan H., He L., Liu H.-Y. (2018). Eucalyptusdimers A–C, Dimeric Phloroglucinol–Phellandrene Meroterpenoids from Eucalyptus robusta. Org. Lett..

[30] Rojas-Garbanzo C., Zimmermann B.F., Schulze-Kaysers N., Schieber A. (2017). Characterization of Phenolic and Other Polar Compounds in Peel and Flesh of Pink Guava (*Psidium Guajava* L. Cv.‘Criolla’) by Ultra-High Performance Liquid Chromatography with Diode Array and Mass Spectrometric Detection. Food Res. Int..

[31] Brezáni V., Šmejkal K. (2013). Secondary Metabolites Isolated from the Genus *Eucalyptus*. Curr. Trends Med. Chem..

[32] Gurbuz P., Baran M.Y., Demirezer L.O., Guvenalp Z., Kuruuzum-Uz A. (2018). Phenylacylated-Flavonoids from Peucedanum chryseum. Rev. Bras. Farmacogn..

[33] Yamakoshi Y., Murata M., Shimizu A., Homma S. (1992). Isolation and Characterization of Macrocarpals BG Antibacterial Compounds from Eucalyptus Macrocarpa. Biosci. Biotechnol. Biochem..

[34] Hasegawa T., Takano F., Takata T., Niiyama M., Ohta T. (2008). Bioactive Monoterpene Glycosides Conjugated with Gallic Acid from the Leaves of Eucalyptus Globulus. Phytochemistry.

[35] Ito H., Koreishi M., Tokuda H., Nishino H., Yoshida T. (2000). Cypellocarpins A−C, Phenol Glycosides Esterified with Oleuropeic Acid, from Eucalyptus Cypellocarpa. J. Nat. Prod..

[36] Chen C., Zhang H., Xiao W., Yong Z.-P., Bai N. (2007). High-Performance Liquid Chromatographic Fingerprint Analysis for Different Origins of Sea Buckthorn Berries. J. Chromatogr. A.

[37] González-Burgos E., Liaudanskas M., Viškelis J., Žvikas V., Janulis V., Gómez-Serranillos M.P. (2018). Antioxidant Activity, Neuroprotective Properties and Bioactive Constituents Analysis of Varying Polarity Extracts from Eucalyptus Globulus Leaves. J. Food Drug Anal..

[38] Al-Sayed E., Martiskainen O., Bobrowska-Hägerstrand M., Sinkkonen J., Törnquist K., Pihlaja K., Ayoub N., Singab A.-N. (2010). Phenolic Compounds from Eucalyptus Gomphocephala with Potential Cytotoxic and Antioxidant Activities. Nat. Prod. Commun..

[39] Saraf I., Marsh K.J., Vir S., Foley W.J., Singh I.P. (2017). Quantitative Analysis of Various B-ring Unsubstituted and Substituted Flavonoids in Ten Australian Species of Eucalyptus. Nat. Prod. Commun..

[40] Wollenweber E., Kohorst G. (1981). Epicuticular Leaf Flavonoids from Eucalyptus Species and from Kalmia Latifolia. Zeitschrift Nat. C.

[41] Ghareeb M.A., Sobeh M., El-Maadawy W.H., Mohammed H.S., Khalil H., Botros S., Wink M. (2019). Chemical Profiling of Polyphenolics in Eucalyptus Globulus and Evaluation of Its Hepato–Renal Protective Potential against Cyclophosphamide Induced Toxicity in Mice. Antioxidants.

[42] Abu-Reidah I.M., Ali-Shtayeh M.S., Jamous R.M., Arráez-Román D., Segura-Carretero A. (2015). HPLC–DAD–ESI-MS/MS Screening of Bioactive Components from Rhus Coriaria L. (Sumac) Fruits. Food Chem..

[43] Yang X.-W., Guo Q.-M. (2007). Studies on Chemical Constituents in Fruits of Eucalyptus Globulus. China J. Chin. Mater. Med..

[44] Jamal A., Yaacob W., Din L.B. (2008). A Chemical Study on Phyllanthus Reticulatus. J. Phys. Sci..

[45] Moore B.D., Wallis I.R., Palá-Paúl J., Brophy J.J., Willis R.H., Foley W.J. (2004). Antiherbivore Chemistry of Eucalyptus—Cues and Deterrents for Marsupial Folivores. J. Chem. Ecol..

[46] Eyles A., Davies N.W., Mohammed C. (2003). Novel Detection of Formylated Phloroglucinol Compounds (Fpcs) in the Wound Wood of Eucalyptus Globulus and E. Nitens. J. Chem. Ecol..

[47] Ablajan K., Abliz Z., Shang X.Y., He J.M., Zhang R.P., Shi J.G. (2006). Structural Characterization of Flavonol 3, 7-Di-O-Glycosides and Determination of the Glycosylation Position by Using Negative Ion Electrospray Ionization Tandem Mass Spectrometry. J. Mass Spectrom..

[48] Tian L.-W., Zhang Y.-J., Wang Y.-F., Lai C.-C., Yang C.-R. (2009). Eucalmaidins A−E, (+)-Oleuropeic Acid Derivatives from the Fresh Leaves of Eucalyptus Maideni. J. Nat. Prod..

[49] Davis B.D., Brodbelt J.S. (2004). Determination of the Glycosylation Site of Flavonoid Monoglucosides by Metal Complexation and Tandem Mass Spectrometry. J. Am. Soc. Mass Spectrom..

[50] Fathoni A., Saepudin E., Cahyana A., Rahayu D., Haib J. (2017). Identification of Nonvolatile Compounds in Clove (Syzygium aromaticum) from Manado. AIP Conference Proceedings.

[51] Tsiri D., Aligiannis N., Graikou K., Spyropoulos C., Chinou I. (2008). Triterpenoids from Eucalyptus Camaldulensis Dehnh. Tissue Cultures. Helv. Chim. Acta.

[52] Wheelan P., Zirrolli J.A., Murphy R.C. (1993). Low-Energy Fast Atom Bombardment Tandem Mass Spectrometry of Monohydroxy Substituted Unsaturated Fatty Acids. Biol. Mass Spectrom..

[53] Kerwin J.L., Wiens A.M., Ericsson L.H. (1996). Identification of Fatty Acids by Electrospray Mass Spectrometry and Tandem Mass Spectrometry. J. Mass Spectrom..

[54] Scott E.N., Gescher A.J., Steward W.P., Brown K. (2009). Development of Dietary Phytochemical Chemopreventive Agents: Biomarkers and Choice of Dose for Early Clinical Trials. Cancer Prev. Res..

[55] Mulyaningsih S., Sporer F., Reichling J., Wink M. (2011). Antibacterial Activity of Essential Oils from Eucalyptus and of Selected Components against Multidrug-Resistant Bacterial Pathogens. Pharm. Biol..

[56] Ghalem B.R., Mohamed B. (2008). Antibacterial Activity of Leaf Essential Oils of Eucalyptus Globulus and Eucalyptus Camaldulensis. Afr. J. Pharm. Pharmacol..

[57] Barbosa J.P., de Oliveira T.R., Puppin D.d.G.P.B., Teixeira A.L., Boni G.C., de Feiria S.N.B., Höfling J.F. (2018). Anti-Candida Activity of Essential Oils from Eucalyptus Species. A Preliminary Study. Oral Health.

[58] Miklasińska-Majdanik M., Kępa M., Wojtyczka R.D., Idzik D., Wąsik T.J. (2018). Phenolic Compounds Diminish Antibiotic Resistance of Staphylococcus Aureus Clinical Strains. Int. J. Environ. Res. Public Health.

[59] Vikram A., Jayaprakasha G.K., Jesudhasan P., Pillai S., Patil B. (2010). Suppression of Bacterial Cell–Cell Signalling, Biofilm Formation and Type Iii Secretion System by Citrus Flavonoids. J. Appl. Microbiol..

[60] Sarkisian S.A., Janssen M., Matta H., Henry G., LaPlante K.L., Rowley D.C. (2012). Inhibition of Bacterial Growth and Biofilm Production by Constituents from *Hypericum* spp.. Phytother. Res..

[61] Monte J., Abreu A.C., Borges A., Simões L.C., Simões M. (2014). Antimicrobial Activity of Selected Phytochemicals against Escherichia Coli and Staphylococcus Aureus and Their Biofilms. Pathogens.

[62] Kim Y.-G., Lee J.-H., Gwon G., Kim S.-I., Park J.G., Lee J. (2016). Essential Oils and Eugenols Inhibit Biofilm Formation and the Virulence of Escherichia Coli O157: H7. Sci. Rep..

[63] Shou Q., Smith J.E., Mon H., Brkljača Z., Smith A.-S., Smith D.M., Griesser H.J., Wohlmuth H. (2014). Rhodomyrtals A–D, Four Unusual Phloroglucinol-Sesquiterpene Adducts from Rhodomyrtus Psidioides. RSC Adv..

[64] Hou A., Liu Y., Lin Z.W., Sun H. (1998). Eucaglobulin, a New Complex of Gallotannin and Monoterpene from Eucalyptus Globulus. Chin. Chem. Lett..

[65] Hou A.-J., Liu Y.-Z., Yang H., Lin Z.-W., Sun H.-D. (2000). Hydrolyzable Tannins and Related Polyphenols from Eucalyptus Globulus. J. Asian Nat. Prod. Res..

[66] Boulekbache-Makhlouf L., Meudec E., Chibane M., Mazauric J.-P., Slimani S., Henry M., Cheynier V., Madani K. (2010). Analysis by High-Performance Liquid Chromatography Diode Array Detection Mass Spectrometry of Phenolic Compounds in Fruit of Eucalyptus Globulus Cultivated in Algeria. J. Agric. Food Chem..

[67] Miranda I., Lima L., Quilhó T., Knapic S., Pereira H. (2016). The Bark of Eucalyptus Sideroxylon as a Source of Phenolic Extracts with Anti-Oxidant Properties. Ind. Crop. Prod..

[68] Luís Â., Silva F., Sousa S., Duarte A.P., Domingues F. (2014). Antistaphylococcal and Biofilm Inhibitory Activities of Gallic, Caffeic, and Chlorogenic Acids. Biofouling.

[69] Bakkiyaraj D., Nandhini J.R., Malathy B., Pandian S.K. (2013). The Anti-Biofilm Potential of Pomegranate (*Punica Granatum* L.) Extract Against Human Bacterial and Fungal Pathogens. Biofouling.

[70] Quave C.L., Estévez-Carmona M., Compadre C.M., Hobby G., Hendrickson H., Beenken K.E., Smeltzer M.S. (2012). Ellagic Acid Derivatives from Rubus Ulmifolius Inhibit Staphylococcus Aureus Biofilm Formation and Improve Response to Antibiotics. PLoS ONE.

[71] Kang M.-S., Oh J.-S., Kang I.-C., Hong S.-J., Choi C.-H. (2008). Inhibitory Effect of Methyl Gallate and Gallic Acid on Oral Bacteria. J. Microbiol..

[72] Shao D., Li J., Li J., Tang R., Liu L., Shi J., Huang Q., Yang H. (2015). Inhibition of Gallic Acid on the Growth and Biofilm Formation of Escherichia Coli and Streptococcus Mutans. J. Food Sci..

[73] Hancock V., Dahl M., Vejborg R.M., Klemm P. (2010). Dietary Plant Components Ellagic Acid and Tannic Acid Inhibit Escherichia Coli Biofilm Formation. J. Med. Microbiol..

